# Betaine and Isoquinoline Alkaloids Protect against Heat Stress and Colonic Permeability in Growing Pigs

**DOI:** 10.3390/antiox9101024

**Published:** 2020-10-21

**Authors:** Hieu Huu Le, Majid Shakeri, Hafiz Ansar Rasul Suleria, Weicheng Zhao, Rachel Mai McQuade, Deborah Jayne Phillips, Eva Vidacs, John Barton Furness, Frank Rowland Dunshea, Valeria Artuso-Ponte, Jeremy James Cottrell

**Affiliations:** 1 Faculty of Veterinary and Agricultural Sciences, The University of Melbourne, Parkville, VIC 3010, Australia; mshakeri@uw.edu (M.S.); hafiz.suleria@unimelb.edu.au (H.A.R.S.); weichengz@student.unimelb.edu.au (W.Z.); dphillips@student.unimelb.edu.au (D.J.P.); vidacse@student.unimelb.edu.au (E.V.); j.furness@unimelb.edu.au (J.B.F.); fdunshea@unimelb.edu.au (F.R.D.); 2 Faculty of Animal Sciences, Vietnam National University of Agriculture, Trau Quy, Gia Lam, Hanoi 131004, Vietnam; 3 Florey Institute of Neuroscience and Mental Health, The University of Melbourne, Parkville, VIC 3010, Australia; rachel.mcquade@unimelb.edu.au; 4 Department of Anatomy and Neuroscience, The University of Melbourne, Parkville, VIC 3010, Australia; 5 Faculty of Biological Sciences, The University of Leeds, Leeds LS2 9JT, UK; 6 Phytobiotics Futterzusatzstoffe GmbH, D-65343 Eltville, Germany; v.artuso@phytobiotics.com

**Keywords:** heat stress, alkaloids, betaine, antioxidants, permeability, gut health, thermoregulation

## Abstract

Heat stress (HS) compromises productivity of pork production, in part as a result of increased oxidative stress and inflammatory responses, particularly within the gastrointestinal tract. This study aimed to investigate whether plant-derived betaine and isoquinoline alkaloids could ameliorate HS in pigs. Fifty female Large White × Landrace grower pigs, which were acclimated to control (CON), control plus betaine (BET), or control plus isoquinoline alkaloids (IQA) diets for 14 days were then exposed to heat stress or thermoneutral condition. Both BET and IQA partially ameliorated increases in respiration rate (*p* = 0.013) and rectal temperature (*p* = 0.001) associated with HS conditions. Heat stress increased salivary cortisol concentrations and reduced plasma creatinine, lactate, and thyroid hormone concentrations. Heat stress increased colon FD4 permeability, which was reduced by IQA (*p* = 0.030). Heat stress increased inflammation in the jejunum and ileum, as indicated by elevated interleukin-1β (*p* = 0.022) in the jejunum and interleukin-1β (*p* = 0.004) and interleukin-8 (*p* = 0.001) in the ileum. No differences in plasma total antioxidant capacity (TAC) were observed with HS, but betaine increased plasma TAC compared to IQA. Dietary BET increased betaine concentrations in the jejunum, ileum (*p* < 0.001 for both), plasma, liver, kidney (*p* < 0.010 for all), urine (*p* = 0.002) and tended to be higher in muscle (*p* = 0.084). Betaine concentration was not influenced by HS, but it tended to be higher in plasma and accumulated in the liver. These data suggest that betaine and isoquinoline alkaloids supplementation ameliorated consequences of heat stress in grower pigs and protected against HS induced increases in colonic permeability.

## 1. Introduction

Pigs are sensitive to heat stress (HS) as they lack functional sweat glands to facilitate heat loss via an evaporative pathway from the skin. Furthermore, the improvement in genetic selection for growth and carcass traits has led to increased metabolic heat production, which decreases the heat tolerance ability of pigs. Heat stress compromises efficient pork production, in part through reductions in feed intake. However, pair feeding studies in other species have shown that only half of the lost productivity can be explained by reductions in feed intake alone [1]. The reasons for this further loss in productivity are multi-faceted but include factors such as disruption of the lining of the gastrointestinal tract (GIT). This arises because a major thermoregulatory mechanism to increase environmental heat loss during hyperthermia is to redistribute blood flow from the visceral organs to the respiratory tract and skin [2,3,4]. The resultant reduced blood flow to the GIT is insufficient to meet metabolic needs and precipitates oxidative stress and loss of the villus epithelium, exposing the lamina propria [5,6]. This results in increased permeability to endotoxins and inflammation [7,8].

With the increasing incidence and severity of heatwaves, there is a focus on developing improved amelioration strategies to counter heat stress and one such approach is through optimizing nutrition [9]. Perhaps the best-known supplement is the organic osmolyte betaine, which has a number of properties that counteract the effects of HS. These include that as an osmolyte betaine reduces the activity of membrane-bound ATPases [10], which may contribute to reducing basal metabolic rate and rectal temperatures in heat-stressed animals [11,12]. Additional properties as a methyl donor could contribute to a wide range of biological reactions reducing oxidative stress and methylation pathways, improving efficiency in dairy [13], meat [14] and egg production [15] from heat-stressed livestock. Plant-derived isoquinoline alkaloids such as sanguinarine reduce stress and *Salmonella* shedding in transported pigs [16,17]. Isoquinoline alkaloids inhibit cellular Na^+^/K^+^ ATPases, which are important contributors to thermogenesis [18,19] and therefore may provide an approach to mitigate heat stress. Additionally, isoquinoline alkaloids have antioxidant [20] and anti-inflammatory properties [20,21] which may counteract oxidative stress, inflammation, and disruption of the GIT mucosa prevalent during HS. Thus, this experiment aimed to investigate whether the supplementation of isoquinoline alkaloids and betaine could ameliorate thermoregulatory responses and protect the intestinal integrity of grower pigs experiencing HS.

## 2. Materials and Methods

### 2.1. Animals and Experimental Design

All experimental procedures were approved by the Animal Ethics Committee of the Faculty of Veterinary and Agricultural Sciences, the University of Melbourne, Australia (Protocol no. 1814434.1) and followed the regulation in the Australian Code for the Care and Use of Animals for Scientific Purposes (8th edition) (National Health and Medical Research Council, 2013).

A total of 50 female grower pigs (Large White × Landrace, 27.3 ± 1.7 kg, mean ± SD) were randomly allocated to one of three experimental diets: a control (CON, *n* = 18, standard grower diet), the CON plus 1 g of betaine/kg of feed (BET, *n* = 16) or the CON with 0.15 g/kg of an isoquinoline alkaloid extract (Sangrovit^®^ Extra, Phytobiotics Futterzusatzstoffe GmbH, Eltville, Germany)/kg of feed (IQA, *n* = 16). Sangrovit^®^ Extra is an extract prepared from *Macleaya cordata* that contains a mixture of isoquinoline alkaloids. It has been previously shown that the principle alkaloids present in capsules of *Macleaya cordata* (mg/g dry weight) were sanguinarine (32.08), chelerythrine (7.36), and dihydrosanguinarine (3.14) while protopine (7.93), allocryptopine (6.77) and sanguinarine (4.51) were major alkaloids found in the aerial part [21]. Furthermore, *Macleaya cordata* contained dihydrochelerythrine (1.1) and trace concentrations of sanguilutine, homochelidonine, norsanguinarine, oxysanguinarine, and oxychelerythrine [21]. Pigs were acclimated to diets for 14 days under thermoneutral (TN) conditions (20 °C; 35–50% relative humidity). The wheat and canola meal based CON diet was formulated to meet the nutrient requirement recommended by the National Research Council (2012) (Table 1). The CON diet contained 13.4 MJ of ME per kilogram and 19.0% of crude protein. The IQA diet contained a feed additive preparation which provided about 1–1.5% of IQA. After the acclimation period half of the pigs from each diet were either continued to be housed under TN conditions or “heat stress” (HS) conditions, which consisted of 8 h/day of 35 °C (0900 to 1700 h) and 28 °C for 16 h/d (1700 to 0900 h) for 3 days (*n* = 25 for both TN and HS). To eliminate effects of dissimilar feed intake all pigs were pair-fed at 2.5 times × ME, which is approximately 75% of ad libitum intake [22] and water was provided ad libitum.

### 2.2. Physiological Observation

Heat stress in the pigs was assessed by daily monitoring of respiration rate (RR), rectal and skin temperature (RT and ST respectively) every 2 h during the heat period (0900, 1100, 1300, 1500, and 1700 h) for days 1 and 2 of the environmental challenge. The RR was observed by counting flank movements for 20 s using a stopwatch, then expressed in breaths/min. Rectal temperature was measured with a digital thermometer (Surgipack, Medtronic Australasia Pty Ltd., Richmond, VIC, Australia) inserted into the rectum approximately 2 cm until the temperature stabilised and skin temperature was recorded on the flank using a hand-held infrared thermometer (Cat no. QM7221, Non-contact IR thermometer, Digitech).

### 2.3. Saliva Collection and Salivary Cortisol Measurement

Saliva was collected by swab (Salivette^®^ Cortisol, Sarstedt AG & Co, Nümbrecht, Germany) at 1500 h on days 1 and 2 of the environmental challenge. The swabs then were centrifuged (1000× *g* at room temperature) for 2 min to obtain saliva, which was stored at −80 °C until analysis. Cortisol concentrations in saliva samples were measured by enzyme-linked immunosorbent assay (ELISA) using commercial kits as per the manufacturer’s instructions (Cat no. 1-3002-5, Salimetrics LLC, State College, PA, USA).

### 2.4. Blood and Tissue Collection, Blood Gas Measurement and Euthanasia

On day 3 of the environmental challenge, pigs were anesthetised with an injection of ketamine (20 mg/kg) and xylazine (3 mg/kg). Once anesthetised, 1 mL samples of fresh blood were collected from the auricular vein and transferred to a blood gas analyser (EPOC^®^; Alere, Waltham, MA, USA). A second blood sample (5 mL) was then collected from the external jugular vein using a vacutainer with EDTA anti-coagulant (BD Australia, North Ryde, NSW, Australia). Plasma was collected after centrifugation at 1500× *g* at 4 °C for 10 min. Plasma samples were stored at −20 °C until analysis.

### 2.5. Plasma Hormones and Metabolites Measurement

Plasma free triiodothyronine (T_3_) and thyroxine (T_4_) were measured with radioimmunoassay kits as per the manufacturer’s instructions (Cat no. 07-221105, MP Biomedicals, Orangeburg, NY, USA). The sensitivity and intra-assay CV were 0.06 pg/dL and 5.12% for T_3_ and 0.45 pg/dL and 5.12% for T_4_. Total antioxidant capacity (TAC) in the plasma was measured by using a commercial kit as per the manufacturer’s instructions (Cat no. 709001, Cayman Chemical, Ann Arbor, MI, USA). The results were expressed as Trolox equivalent antioxidant capacity with the average inter and intra-assay CV were 2.04% and 1.47% respectively. Likewise, plasma albumin, triglyceride, bilirubin concentrations were quantified using reagents supplied from Cat no. 981767, 981786, 981897 (Thermo Fisher Scientific, Waltham, MA, USA) as per manufacturer’s instructions. The inter- and intra-assay CVs of 9.4% and 11.9% for albumin, 3.8% and 2.1% for triglycerides, and 4.9% and 2.0% for bilirubin.

### 2.6. Urinalysis

Urine samples were collected directly from the bladder soon after euthanasia using a transmural needle and syringe. The pH was measured immediately (Eutech pH 5+ pH meter, Thermo Fisher Scientific, Waltham, MA, USA) and osmolality was quantified (Advanced Micro Osmometer 3300, Advanced Instruments, Norwood, MA, USA). Urinary albumin and bilirubin concentration were quantified using commercial reagents (Cat no. 981767, 981897, Thermo Fisher Scientific, Waltham, MA, USA) as per manufacturer’s instructions, and the inter- and intra-assay variations of 7.5% and 0.9% for albumin and 8.4% and 2.7% for bilirubin. Urinary creatinine concentration was quantified using a commercial kit as per the manufacturer’s instructions (Cat no. 500701, Cayman Chemical, Ann Arbor, MI, USA), with the inter- and intra-assay CV% being 3.1% and 1.0%, respectively.

### 2.7. Tissue Sampling and Intestinal Permeability Measurement

Sections of distal ileum (~30 cm proximal to ileocecal junction) and proximal colon (immediately distal to ileocecal junction) were collected immediately after euthanasia and placed in chilled phosphate-buffered saline (0.9% Sodium Chloride Intravenous, Baxter Healthcare, Old Toongabbie, NSW, Australia), then transferred to Krebs solution. The mucosa was removed by blunt dissection and mounted onto a round slider and placed into a two-part Ussing chamber (EasyMount Diffusion Chambers, Physiologic Instruments, San Diego, CA, USA) as described by [23]. Afterwards, 200 µL 25 mg/mL fluorescein isothiocyanate-dextran (FD4) (Sigma-Aldrich, Saint Louis, MO, USA) was added into the mucosal side of the chamber, and 100 µL solution from the serosal side of a chamber were collected at 0, 60, and 120 min for quantifying mucosa FD4 apparent permeation coefficient (*P_app_*) in duplicate by the following equation [24]:

$$P_{app}\;=\;\frac{dQ}{\left(dt\times A\times C_{0}\right)}$$

where *dQ*/*dt* is the transport rate (in µg/s) and corresponds to the linear slope of the three measures, *C*_0_ is the initial concentration in donor chamber, and *A* is the area of the slider (0.71 cm^2^).

### 2.8. Measurement of Inflammation Markers by ELISA

Frozen jejunum and ileum samples were pulverized under liquid nitrogen, then 100 mg of frozen tissue homogenized in 1 mL of chilled buffer (Cat no. 89901, Thermo Fisher Scientific, Waltham, MA, USA) then centrifuged at 10,000× *g* for 15 min at 4 °C. The supernate was collected and frozen at −20 °C for subsequent ELISA of interleukin-1β (IL-1β), interleukin-8 (IL-8), and tumor necrosis factor-alpha (TNF-α) concentrations using commercial kits as per the manufacturer’s instructions (Cat no. DY681, DY535, and DY690B, R&D Systems, Minneapolis, MI, USA).

### 2.9. Betaine Distribution

Betaine concentrations were quantified in plasma, urine, liver, kidney, muscle, jejunum, ileum, and colon tissue. Plasma concentrations were determined by high-pressure liquid chromatography method (HPLC) as described by [25] with modifications described by [14]. Tissue samples were first snap frozen and then pulverized in liquid nitrogen. One hundred mg of pulverized tissue was then homogenized in 1 mL of cold TRIS buffer (1 M, pH 7.0) using a bead beater (Mini-BeadBeater-8, BioSpec Products, Bartlesville, OK, USA) for 1 min. The sample was then centrifuged at 14,400× *g* for 20 min at 4 °C. The supernate was then collected for assay as per plasma samples with results expressed as mg betaine/g tissue.

### 2.10. Test Diet Sanguinarine Concentration

One gram of feel was crushed with a mortar and pestle and extracted in 15 mL of 95:5 acetonitrile:water for 30 min with sonication then centrifuged for 10 min at 10,000× *g*. The supernate was collected mixed with 10 mL of n-hexane then centrifuged at 10,000× *g* for 3 min. The supernatant was removed and dried under nitrogen gas at 30 °C. The residue of the supernate was reconstituted in 5 mL methanol and 15 mL of 1% hydrochloric acid-methanol (10:90 *v*/*v*) was added to the pellet and the processes above repeated before reconstitution with 5 mL methanol. The resulting methanol solutions were mixed and centrifuged at 12,000× *g* for 10 min then filtered through a 0.22 mm filter before assay by LCMS as described by [26] using Sanguinarine chloride hydrate (>98% Sigma Aldrich, St Louis, MO, USA). The concentration of Sanguinarine in the supplemented diet was 0.61 mg/kg feed.

### 2.11. Statistical Analysis

The experiment comprised a 2 × 3 factorial design, therefore parameters were analysed for the pooled main effects of temperature (TN vs. HS), diet (CON, BET, IQA), and their respective interactions using an unbalanced ANOVA (Genstat v. 18, VSN International, Hemel Hempstead, UK). Where appropriate time was included in the model and the experimental replicate (*n* = 5) was used as a blocking factor. When skewed data were present, as was the case for urinary betaine and salivary cortisol, the validity of the ANOVA was confirmed by repeating the analysis following a Log_10_ transformation which reduced the heterogeneity of variances. Values presented in the text are for the adjusted means and standard error of the difference (SED) for the pooled main effects of temperature (T), diet (D), or time, while values presented in figures and tables are for the full interactions (T × D, T × D × time). For the ANOVA groups were considered significantly different at *p* ≤ 0.05, while a trend was considered at *p* ≤ 0.10. A post-hoc Fisher’s least significant difference test was used to compared multiple means, with alphabetical superscripts used to identify different groups at *p* < 0.05.

## 3. Results

### 3.1. Physiological Responses

**Figure 1:**
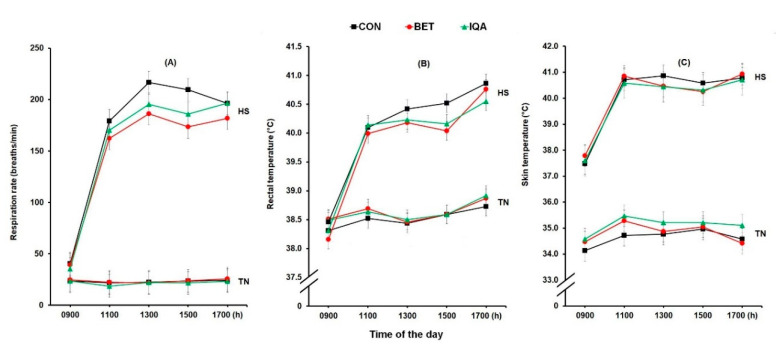
Changes in daily respiration rate (**A**), rectal temperature (**B**), and skin temperature (**C**) of grower pigs fed a control diet (CON), betaine (BET), or isoquinoline alkaloids (IQA) diets under thermoneutral (TN) or heat stress conditions (HS). Each figure represents the adjusted means ± pooled SED of two days of observations for the interaction between T × D × time. Panel (**A**) the respiration rate was significantly increased by HS (*p* < 0.001), time of the day (*p* < 0.001) and was lower with BET and IQA than CON overall (diet *p* = 0.024) and during HS (diet*temp. *p* = 0.013). (**B**) Rectal temperature was significant increased by HS (*p* < 0.001), time of the day (*p* < 0.001) and was lower with BET and IQA than CON during HS (diet *p* = 0.001) while no main effect of diet was observed. (**C**) Skin temperature was increased by HS (*p* < 0.001), time of the day (*p* < 0.001) and was lower with IQA than CON during HS (diet *p* = 0.046) while no main effect of diet was observed. There were interactions between temp × time (*p* < 0.001) on respiration rate, rectal temperature and skin temperature whereas no temp × diet × time was observed and each group represents *n* = 8–9 pigs.

Respiration rate increased in response to HS (23 vs. 159 breaths/min, *p* < 0.001) and increased over the time of day (31^a^, 98^b^, 109^c^, 110^c^ and 113^c^ breaths/min at 0900, 1100, 1300, 1500, and 1700 h, respectively; *p* < 0.001; Figure 1A). There was an interaction between environmental condition and time of the day (*p* < 0.001) such that RR was increased to a greater extent over the day in pigs subjected to HS compared to those housed in TN condition (Figure 1A). There was a significant effect of dietary treatment on RR such that pigs fed BET or IQA had lower RR than CON (98^a^, 88^b^, and 91^b^ breaths/min for CON, BET, and IQA, respectively; *p* = 0.024). Furthermore, there was also a significant interaction between HS and diet (*p* = 0.013) such that RR were lower in pigs given BET or IQA and subjected to HS but not in those housed in TN condition (Figure 1A).

Rectal temperature was increased by HS (38.6 vs. 39.9 °C, *p* < 0.001) and by time of the day (38.4^a^, 39.3^b^, 39.4^b^, 39.4^b^, and 39.8^c^ °C at 900, 1100, 1300, 1500, and 1700 h, respectively; *p* < 0.001; Figure 1B). However, there was an interaction between environmental condition and time (*p* < 0.001) such that the RT of pigs exposed to HS increased to a greater extent in comparison with those subjected to TN condition (Figure 1B). Although there was no main effect of dietary treatment on RT, there was an interaction between HS and diet (*p* = 0.001) such that RT were lower in pigs fed pigs fed BET or IQA and exposed to HS but not in the pigs kept in TN condition (Figure 1B).

Similar effects were observed on skin temperature. Skin temperature was increased by HS (34.9 vs. 40.0 °C, *p* < 0.001) and by time of the day (36.0^a^, 37.7^b^, 37.8^b^, 37.8^b^, and 37.9^b^ °C at 0900, 1100, 1300, 1500, and 1700 h, respectively; *p* < 0.001; Figure 1C). However, there was an interaction between environmental condition and time of heat exposed day (*p* < 0.001) such that pigs subjected to HS had higher ST at 1100, 1300, 1500, and 1700 h than at 0900 h while other times were not different. Although no main effect of dietary treatment on ST was observed, there was an interaction between temperature and diet (*p* = 0.046) such that ST was higher in pigs fed IQA than CON in TN condition, while all other groups were not different.

### 3.2. Blood Oximetry and Electrolytes

Blood pH decreased in response to HS (7.45 vs. 7.43, *p* = 0.013, Table 2) but remained unchanged with dietary treatment and interaction between environmental condition and diet. Similarly, blood partial of oxygen (_P_O_2_) reduced in response to HS (67.2 vs. 46.6 mmHg, *p* < 0.001). There were no main effects of diet or interactive effect on _P_O_2_. Blood O_2_ saturation was decreased by HS (93.3 vs. 82.0%, *p* < 0.001) and tended to be lowered by IQA compared to CON (89.3, 88.8, and 86.2% for CON, BET and IQA, respectively; *p* = 0.096). However, there was an interaction between environmental conditions and diet (*p* = 0.035) such that pigs fed IQA had lower blood O_2_ saturation compared with those fed CON and BET under HS (85.2, 81.9, and 78.2% for CON, BET, and IQA, respectively) but not under TN condition. Although blood partial pressure of carbon dioxide (_P_CO_2_) was not influenced by HS, BET tended to lower this parameter compared to CON and IQA (48.6, 46.5, and 48.7 mmHg for CON, BET, and IQA, respectively; *p* = 0.096). Heat stress tended to reduce the concentration of total blood carbon dioxide (_T_CO_2_, 34.8 vs. 33.6 mM, *p* = 0.074) and bicarbonate (HCO_3_, 33.3 vs. 32.1 mM, *p* = 0.054) whereas there were no dietary or interactive effects. Blood lactate decreased in response to HS (1.89 vs. 1.15 mM, *p* = 0.001) but there were no main or interactive effect of diets. Base excess in blood (BE_b_) was decreased by HS (8.16 vs. 6.97 mM, *p* = 0.029). Although there was no main effect of dietary treatment, there was an indication of an interaction (*p* = 0.088) between environmental condition and diet such that BE_b_ declined to a greater extent in pigs fed BET or IQA compared to CON (8.29, 6.23 and 6.38 mM for CON, BET and IQA, respectively) during HS. Similar effects of those were seen for base excess in extracellular fluid (BE_ecf_, Table 2). Base excess in extracellular fluid decreased in response to HS (9.36 vs. 8.01 mM, *p* = 0.032). While there was no main effect of diet, there was an indication of an interaction (*p* = 0.098) between environmental condition and diet such that BE_ecf_ of pigs fed BET or IQA decreased to a greater extent compared to those in CON (9.54, 7.14 and 7.35 mM for CON, BET and IQA, respectively) under HS condition. Blood concentration of potassium increased in response to HS (4.05 vs. 4.30 mM, *p* = 0.001) but no main effect of diet or interaction was observed. Similarly, blood chloride concentration was increased by HS (98.4 vs. 100.4 mM, *p* = 0.027) but there was no main effect of diet or interactive effect. There was no effect of environmental condition or dietary treatment on blood concentration of sodium, calcium, anion gap, and glucose (Table 2). Heat stress decreased blood haematocrit (35.3 vs. 31.8%, *p* < 0.001) and haemoglobin concentration (11.9 vs. 10.8 g/dL, *p* < 0.001) but there were no dietary or interactive effects on these parameters (Table 2).

### 3.3. Blood and Urine Biochemistry

Plasma creatinine increased in response to HS (0.126 vs. 0.146 mM, *p* < 0.001, Table 3) but was unaffected by dietary treatment. Heat stress had no effect on plasma albumin concentration but there was a main effect of diet such that this pigs in BET and IQA diet had lower the albumin concentration than those in CON (44.4, 39.9, and 40.1 g/L for CON, BET and IQA, respectively; *p* = 0.048). There were no main effects of environmental condition and dietary treatment or interaction on plasma protein, bilirubin and triglyceride concentration (Table 3).

Urinary osmolality was increased by HS (163 vs. 334 mOsm/kg H_2_O, *p* = 0.007, Table 4) but unchanged with dietary treatment. Similarly, HS increased urinary concentration of albumin (0.399 vs. 0.672 g/L, *p* < 0.001) and creatinine (3.39 vs. 8.57 mM, *p* = 0.002, Table 4) but there were no dietary or interactive effects on these parameters. The concentration of bilirubin increased in response to HS (7.98 vs. 16.3 µM, *p* = 0.014). While no significant effect of diet was observed, there was an interaction between HS and diet (*p* = 0.030) such that urinary concentration was lower in pigs fed IQA than those fed BET and CON in HS condition (17.7, 21.0, and 5.65 µM for CON, BET, and IQA, respectively) (Table 4).

### 3.4. Endocrine Responses to HS

**Figure 2:**
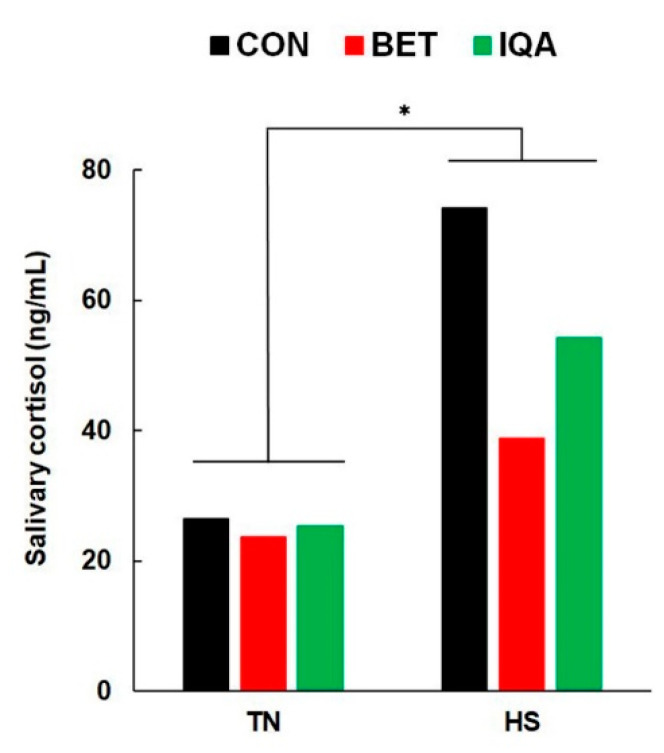
Effect of thermoneutral (TN) and heat stress (HS) conditions on salivary cortisol concentrations of growing pigs fed a control diet (CON), the control plus betaine (BET), or the control plus isoquinoline alkaloids (IQA). Each bar represents the pooled effect of saliva collected on days 1 and 2 of the heat challenge. Due to sample heterogeneity, the results were transformed (log_10_) before analysis, then presented as mean of back-transformed means. HS significantly increased salivary cortisol (* *p* = 0.010). No effects of diet or interactions with temperature were observed. *n* = 8–9 pigs per group, with values representing adjusted means ± pooled SED for the interaction between T × D.

Salivary cortisol increased in response to HS (25.2 vs. 54.6 ng/mL, *p* = 0.010, Figure 2). There were no main (*p* = 0.56) or interactive (*p* = 0.75) effects of dietary treatment on salivary cortisol. Plasma-free T_3_ decreased in response to HS (0.66 vs. 0.47 pg/mL, *p* < 0.001, Table 3) but there were no main effects of diet or interaction. Similarly, plasma-free T_4_ decreased in response to HS (15.4 vs. 9.55 pg/mL, *p* < 0.001, Table 3). Dietary treatment had a significant effect on plasma free T_4_ such that this parameter was lower in pigs fed IQA than those in CON and BET (13.6^a^, 12.5^a^, and 11.2^b^ ng/dL for CON, BET, and IQA, respectively; *p* = 0.033, Table 3). There was no interactive effect between environmental condition and diet on plasma free T_4_.

### 3.5. Inflammatory and Antioxidant Markers

Interleukin-1β concentrations increased in response to HS in both the jejunum (125 vs. 193 pg/mL, *p* = 0.002, Table 5) and ileum (299 vs. 414 pg/mL, *p* = 0.004). Although there was no main effect of diet, there was an indication of an interaction between environmental condition and diet on jejunal IL-1β (*p* = 0.056) such that HS pigs on BET tended to increase IL-1β concentration compared to those on CON and IQA (173, 253, and 154 pg/mL for CON, BET and IQA, respectively). Interleukin-8 concentrations were increased by HS in the ileum (2639 vs. 3108 pg/mL, *p* = 0.012) but unchanged in the jejunum. There were no main or interactive dietary effects on IL-8. There were no effects of HS or diet on TNF-α (Table 5). Erythrocyte sedimentation rate (ESR) was increased by HS (9.4 vs. 12.5 mm/h, *p* = 0.021, Table 5) but unchanged by diets. There was no main effect of environmental conditions on plasma TAC. However, there was a significant effect of the dietary treatment such that TAC was higher in BET than IQA, but neither diet was significantly different from CON (1.36^a^, 1.62^ab^, 1.19^ac^ mM Trolox for CON, BET, and IQA, respectively; *p* = 0.020, Table 5).

### 3.6. Intestinal Permeability

**Figure 3:**
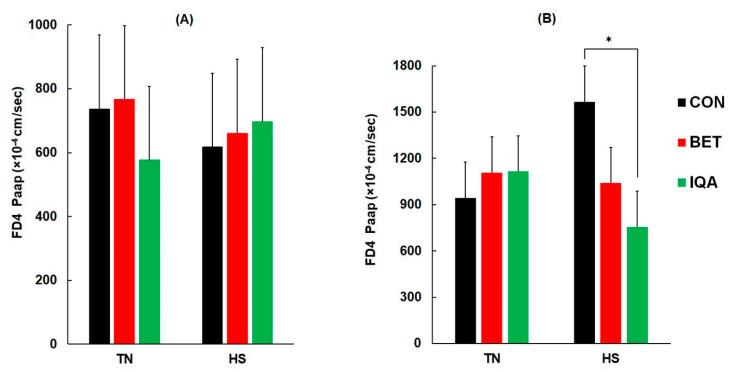
Effect of heat stress (HS) and thermoneutral (TN) conditions on ileal (**A**) and colonic (**B**) fluorescein isothiocyanate dextran (4 k Da) apparent permeation coefficient (FD4 *P_app_*) of growing pigs fed control diet (CON), the control plus betaine (BET) or the control plus isoquinoline alkaloids (IQA). Results are adjusted mean ± SED and *n* = 8–9 pigs per group. (**A**) The *p*-value for the effects of temp., diet and diet*temp were 0.77, 0.90 and 0.71 respectively. (**B**) The *p*-value for the effects of temp., diet and diet*temp were 0.56, 0.23 and 0.030 respectively. * denotes *p* < 0.05 as determined by Fisher’s least significant difference test.

Ileal permeability was unaffected by temperature or diet (Figure 3A). Although there were no main effects of HS or diet on colonic permeability, there was a significant interaction between temperature and diet (*p* = 0.030) such that FD4 permeability of pigs fed IQA was lower than CON in HS condition (1566, 1040 and 756 × 10^−4^ cm/sec for CON, BET, and IQA, respectively) while all other group were not significant (Figure 3B).

### 3.7. Betaine Distribution

Supplementation with betaine increased betaine concentrations in plasma (840^a^, 1282^b^, and 811^a^ µmol/L for CON, BET, and IQA, respectively, *p* < 0.001, Table 6), urine (1910^a^, 5957^b^, and 1570^a^ µmol/L, *p* = 0.002), liver (1.16^a^, 1.55^b^, and 1.18^a^ mg/g, *p* = 0.007), kidney (1.00^a^, 1.12^b^, and 0.71^a^ mg/g *p* < 0.001), jejunum (0.37^a^, 0.49^b^, and 0.35^a^ and mg/g *p* <0.001) and ileum (0.47^a^, 0.58^b^, and 0.44^a^ mg/g *p* < 0.001). Betaine concentrations tended to be higher in muscle with BET (0.52, 0.74, and 0.58 mg/g, *p* = 0.080), but no increases in colon betaine concentrations were observed. Irrespective of diet betaine concentrations of HS pigs tended to be higher in plasma (923 vs. 1021 µmol/L, *p* = 0.08) and the liver (1.20 vs. 1.38 mg/g, *p* = 0.07). Ileal betaine concentration tended to be increased by BET in TN condition (0.45, 0.61, and 0.48 mg/g, interaction *p* = 0.097) but the trend was not observed in HS.

## 4. Discussion

The key findings of this study were that supplementation with betaine and isoquinoline alkaloids partially ameliorated the thermoregulatory responses to HS in grower pigs. This was evidenced by the combination of a reduction in RR coupled with a lower RT. The significance of this finding is that as pigs lack water producing sweat glands [27] they, therefore, rely on “evaporative panting” during HS to increase heat loss to the environment. In this process, blood flow to the upper respiratory tract is increased and the pattern of respiration altered such that tidal volume is decreased, but the respiration rate [28] and blood flow to the upper respiratory tract are increased [4]. This increases airflow in the upper respiratory tract, enabling cooling by the evaporation of drool. Therefore, these results indicate that BET and IQA diets ameliorated HS, by lowering core body temperature (RT) and also the amount of effort required to lower temperatures, as evidenced by the lower RR.

Reductions in intestinal blood flow during hyperthermia can compromise GIT blood flow, leading to epithelial cell loss and exposure of the lamina propria, which in the ileum has been associated with reduced transepithelial resistance (TER) and increased permeability to macromolecules and bacterial lipopolysaccharide [8,29,30]. Consistent with published data, we found that colonic macromolecular permeability was about 60% greater in HS compared to TN pigs. The colon was responsive to nutritional modification, with IQA preventing the increases in macromolecule permeability that occurred in HS pigs, suggesting improved mucosal integrity. Beneficial effects of sanguinarine on reducing colonic leakiness have been demonstrated elsewhere in a model of colitis in rats [31] and collectively these results support the colon being a site of action for IQA’s. The IQA formulation used is derived from *Macleaya cordata*, otherwise known as “plume poppy”, and is rich in IQA’s such as sanguinarine [21]. Extracts of *Macleaya cordata* have been shown to improve GIT health, as demonstrated by lowering diarrhea scores and *Salmonella* populations whilst promoting mucosal growth [17,20,32]. In a separate experiment, *Macleaya cordata* extracts reduced *Salmonella*, increased *Lactobacillus* populations, antioxidant capacity, parameters of immune function, small intestinal villus height, and growth performance [20].

There appear to be multiple mechanisms whereby IQA can benefit the GIT mucosa. This includes antimicrobial and anthelminthic properties [33]. Moreover, other experiments in sheep have observed that *Macleaya cordata* extracts reduce oxidative stress [34], including in heat-stressed animals where improvements in feed conversion efficiency were observed [35]. Isoquinoline alkaloids are widely reported to have anti-inflammatory properties [31,36], with the effect at least partially mediated via changes in NF-*k*β [37]. While the HS model used in the present experiment increased inflammation in the small intestine and blood ESR, which is also used as a marker of inflammation, no influence of IQA on markers of inflammation was observed. It is noted that sanguinarine has poor bioavailability [38,39,40], but that metabolism by the pig microbiome and intestinal mucosa has been quantified [40]. In that experiment, colonic mucosa was the site of greatest sanguinarine metabolism to dihydroxysanguinarine (DHSA), while the ileal microbiome was the most active region of metabolism by the microbiome. This positions the distal regions of the GIT as the most significant sites of sanguinarine metabolism, which is consistent with the colon being the site of reductions in permeability in the current experiment.

As IQA’s such as sanguinarine have low bioavailability, the reductions in RR and RT were unexpected. Of a single oral dose of 10 mg/kg body weight sanguinarine, only 15% was excreted in the urine, but 17.3% of the dose was retained in the liver, 29% in the GIT, and 3.6% in the kidney [41], showing that some of the sanguinarine is bioavailable and therefore may influence thermoregulation beyond the GIT. There may be important effects within the GIT that contribute to reduced thermogenesis, namely the inhibitory effect on Na^+^/K^+^ ATPase pump activity [18,42]. This pump activity has a substantial contribution to thermogenesis, providing as much as 72% of heat production in animals [19]. Betaine also reduces thermogenesis through lowering Na^+^/K^+^ ATPase activity [10], although the bioavailability of betaine is substantially higher. Additionally, IQAs can reduce fermentation, which also contributes to heat production, in the large intestine [43]. Elsewhere, dietary IQA’s have been demonstrated to have post-absorptive effects, such as improvements in antioxidant capacity, and also reduce cortisol concentrations following transport stress [16], supporting effects beyond the GIT. Considering these results in comparison to those of [38], who showed that only a small alkaloid proportion was absorbed, the effects of sanguinarine and other isoquinoline alkaloids beyond the GIT warrants further investigation.

Like isoquinoline alkaloids, supplementation with betaine ameliorated the increases in RR and RT in the HS grower pigs, closely reflecting results seen in broilers [11,14] and sheep [44]. Betaine has at least three known biological functions, as a methyl donor, an osmolyte, or directly as a chaperone [9]. Metabolism of betaine is primarily as a methyl donor to homocysteine in the methionine cycle. This reaction is catalysed by the enzyme betaine homocysteine methyltransferase (BHMT), which is expressed primarily in the liver and to a lesser degree the kidney [45]. Supplemental betaine increased in the jejunum and ileum, but not the colon, supporting studies showing that betaine has high bioavailability [46,47]. However, despite being elevated in the jejunum and ileum no improvements in mucosal *P_app_* were observed. This differs from the results of [48], who observed reductions in ileal permeability. Importantly, the study by [48] used a dose of 0.125% betaine as opposed to 0.1% in the current study and supports the use of higher doses of betaine to ameliorate HS. Increased distribution of supplemental betaine was observed in the liver and kidneys, which is consistent with other studies [14,46], these organs being key sites of betaine utilisation. In humans, the primary metabolic fate of betaine is metabolism to dimethylglycine [47]. It is noteworthy that in the current experiment there appeared to be significant excretion of unmodified betaine, indicating that a proportion of the effects of betaine in the current experiment were due to direct osmolyte effects such as reducing the activity of Na^+^/K^+^-ATPase and Ca^2+^-ATPases [10] or chaperone effects.

## 5. Conclusions

Both IQA and BET ameliorated heat stress in grower pigs but appear to do so by different mechanisms. Isoquinoline alkaloids appeared to work primarily on the GIT, providing the added benefit of protecting against increases in colonic permeability caused by heat stress. By contrast, betaine appeared to be readily absorbed from the GIT, disappearing from GIT tissue before the colon. Urinary excretion of unmodified betaine indicates that part of the action is linked to its being a cellular osmolyte, but elevated distribution to the liver and kidneys, which are sites of betaine metabolism, was also observed.

## Figures and Tables

**Table 1:** Ingredient and composition of diets as fresh feed basis.

Item	Amount
Ingredient, %	
Wheat	50.5
Barley	7.00
Lupins	15.0
Millmix ^1^	4.00
Canola meal 38%	13.8
Soy bean meal 46%	5.00
Water	2.00
Tallow mixer	0.800
Limestone	1.53
DL-Methionine	0.060
Lysine	0.030
Threonine	0.055
Calculated values	
Digestible energy (MJ/kg)	13.4
Crude protein (%)	19.0
Lysine (%)	1.09
Calcium (%)	0.887
Total phosphorous (%)	0.533

^1^ The amount of Millmix was adjusted to include Sangrovit Extra and betaine in the diets at 0.15 and 1 g/kg respectively. The final concentration of sanguinarine in the test diet was confirmed to be 0.61 mg/kg by assay (Section 2.10).

**Table 2:** Effect of thermoneutral (TN) and heat stress (HS) conditions and control (CON), betaine (BET) or isoquinoline alkaloids (IQA) diets on blood gases and electrolytes ^1^.

Parameter ^2^	TN			HS			SED	*p*-Value		
	CON	BET	IQA	CON	BET	IQA		T	D	T × D
pH	7.44	7.45	7.46	7.44	7.43	7.43	0.01	0.013	0.89	0.21
_p_O_2_ (mmHg)	66.8	68.3	66.8	50.2	44.6	43.9	3.13	<0.001	0.50	0.16
O_2_ saturation (%)	93.0 ^a^	93.7 ^a^	93.3 ^a^	85.2 ^b^	81.9 ^bc^	78.2 ^c^	2.01	<0.001	0.096	0.035
_P_CO_2_ (mmHg)	48.1	46.9	47.9	49.2	46.4	48.5	1.62	0.35	0.096	0.56
Total CO_2_ (mM)	34.6	34.3	35.4	34.6	32.9	33.3	1.10	0.074	0.43	0.37
HCO_3_ (mM)	33.1	32.9	33.9	33.1	31.5	31.8	1.05	0.054	0.44	0.35
Sodium (mM)	142	143	142	143	141	141	1.69	0.59	0.64	0.59
Potassium (mM)	3.95	4.17	4.04	4.38	4.26	4.26	0.13	0.001	0.77	0.15
Chloride (mM)	98.2	98.0	99.1	100	101	99.5	1.53	0.027	0.86	0.35
Calcium (mM)	1.37	1.38	1.37	1.36	1.35	1.40	0.03	0.92	0.51	0.40
Anion gap (mM)	14.8	15.9	14.6	14.6	14.8	14.4	0.72	0.23	0.21	0.64
Glucose (mM)	6.23	6.58	6.26	6.52	5.96	6.81	0.39	0.66	0.68	0.10
Lactate (mM)	1.92	1.86	1.87	1.08	1.27	1.12	0.33	0.001	0.96	0.86
BE_b_ (mM)	7.83	7.89	8.76	8.29	6.23	6.38	0.93	0.029	0.32	0.088
BE_ecf_ (mM)	9.02	9.01	10.0	9.54	7.14	7.35	1.06	0.032	0.29	0.098
Hwt (%)	35.4	35.1	35.3	33.2	30.7	31.2	1.34	<0.001	0.31	0.45
HgB (g/dL)	11.8	11.9	12.0	11.3	10.5	10.6	0.44	<0.001	0.50	0.25

^1^*n* = 8–9 pigs per group with values representing adjusted means ± pooled standard error of the difference (SED) for the interaction between T × D. ^2^ BE_b_: base excess in blood; BE_ecf_: base excess in extra cellular fluid; Hwt: Haematocrit; HgB: Haemoglobin. ^a–c^ Differing superscripts denote groups *p* < 0.05.

**Table 3:** Effect of thermoneutral (TN) and heat stress (HS) conditions and control (CON), betaine (BET) or isoquinoline alkaloids (IQA) diets on plasma biochemistry and hormones ^1^.

Parameter	TN			HS			SED	*p*-Value		
	CON	BET	IQA	CON	BET	IQA		T	D	T × D
Protein (mg/mL)	51.3	54.4	54.1	55.7	53.7	53.2	3.60	0.59	0.98	0.47
Albumin (g/L)	43.0	39.5	39.9	46.0	40.5	40.4	2.87	0.33	0.048	0.78
Bilirubin (µM)	6.01	8.37	6.97	7.01	7.47	5.72	1.22	0.64	0.15	0.37
Creatinine (mM)	0.130	0.129	0.118	0.148	0.139	0.148	0.01	<0.001	0.45	0.16
Free T_3_ (pg/mL)	0.71	0.68	0.58	0.45	0.48	0.50	0.07	<0.001	0.68	0.35
Free T_4_ (pg/mL)	17.0	15.8	13.2	10.1	9.28	9.22	1.38	<0.001	0.03	0.26

^1^*n* = 8–9 pigs per group, with values representing adjusted means ± pooled standard error of the difference (SED) for the interaction between T × D.

**Table 4:** Effect of thermoneutral (TN) and heat stress (HS) conditions and control (CON), betaine (BET) or isoquinoline alkaloids (IQA) diets on urine biochemistry and metabolites ^1^.

Parameter	TN			HS			SED	*p*-Value		
	CON	BET	IQA	CON	BET	IQA		T	D	T × D
pH	5.95	6.44	6.50	6.39	6.23	6.41	0.24	0.67	0.29	0.16
Osmolality (mOsm/kg H_2_O)	130	210	154	406	227	338	98	0.005	0.78	0.18
Albumin (g/L)	0.352	0.404	0.451	0.579	0.627	0.830	0.109	<0.001	0.11	0.53
Bilirubin (µM)	5.78 ^a^	7.91 ^ab^	10.9 ^abc^	17.7 ^bc^	21.0 ^c^	5.65 ^a^	5.16	0.014	0.33	0.030
Creatinine (mM)	1.85	3.69	4.93	9.49	10.3	4.92	2.73	0.002	0.63	0.15

^1^*n* = 8–9 pigs per group, with values representing adjusted means ± pooled standard error of the difference (SED) for the interaction between T × D. ^a–c^ Differing superscripts denote groups *p* < 0.05.

**Table 5:** Effect of thermoneutral (TN) and heat stress (HS) conditions and control (CON), betaine (BET) or isoquinoline alkaloids (IQA) on intestinal inflammatory responses ^1^.

Parameter	TN			HS			SED	*p*-Value		
	CON	BET	IQA	CON	BET	IQA		T	D	T × D
Jejunum										
IL-1β (pg/mL)	130	115	130	173	253	154	34.8	0.002	0.21	0.056
IL-8 (pg/mL)	2965	3301	3026	3470	3044	3011	0.297	0.58	0.62	0.18
TNF-α (pg/mL)	28.2	32.0	30.2	26.6	24.3	27.8	4.38	0.14	0.87	0.57
Ileum										
IL-1β (pg/mL)	228	343	334	402	431	408	62.8	0.004	0.25	0.46
IL-8 (pg/mL)	2611	2706	2604	2983	3111	3247	310	0.012	0.81	0.80
TNF-α (pg/mL)	36.3	35.3	33.2	34.0	39.4	41.2	4.78	0.29	0.75	0.30
ESR (mm/h)	8.3	8.7	7.8	12.2	14.1	11.3	2.10	0.001	0.45	0.83
TAC (mM Trolox)	1.34	1.61	1.17	1.38	1.63	1.21	0.20	0.73	0.020^5^	1.00

^1^*n* = 8–9 pigs per group, with values representing adjusted means ± pooled standard error of the difference (SED) for the interaction between T × D.

**Table 6:** Effect of thermoneutral (TN) and heat stress (HS) conditions and control (CON), betaine (BET) or isoquinoline alkaloids (IQA) on betaine distribution in growing pigs ^1^.

Parameter	TN			HS			SED	*p*-Value		
	CON	BET	IQA	CON	BET	IQA		T	D	T × D
Plasma (µmol/L)	731	791	1271	949	831	1293	98.4	0.088	<0.001	0.28
Urine ^2^	3.23	3.69	3.20	3.33	3.86	3.19	0.22	0.50	0.002	0.83
(µmol/L)	(1714)	(4853)	(1600)	(2128)	(7311)	(1538)				
Tissue (mg/g)										
Muscle	0.50	0.74	0.51	0.54	0.74	0.65	0.14	0.50	0.084	0.80
Liver	0.94	1.54	1.14	1.38	1.56	1.22	0.18	0.070	0.007	0.20
Kidney	0.93	1.19	0.83	1.06	1.05	0.61	0.14	0.42	<0.001	0.21
Jejunum	0.34	0.45	0.36	0.40	0.52	0.33	0.12	0.15	<0.001	0.19
Ileum	0.45	0.61	0.48	0.49	0.51	0.43	0.04	0.28	<0.001	0.097
Colon	0.50	0.48	0.36	0.43	0.55	0.52	0.07	0.51	0.66	0.12

^1^*n* = 8–9 pigs per group, with values representing adjusted means ± pooled standard error of the difference (SED) for the interaction between T × D. ^2^ Data were skewed so were log_10_-transformed before analysis and back-transformed means are presented in parentheses.
